# Contrasting photosynthetic capacity and carbon allocation under increasing irradiance in two *Paris polyphylla* varieties

**DOI:** 10.3389/fpls.2026.1940365

**Published:** 2026-09-04

**Authors:** Feiyan Wen, Hanyi Zhang, Jinyu Liu, Siyu Chen, Jin Pei, Tao Zhou

**Affiliations:** 1 College of Pharmacy, Chengdu University of Traditional Chinese Medicine, Chengdu, Sichuan, China; 2 Chinese Medicine Germplasm Resources Innovation and Effective Uses Key Laboratory of Sichuan Province, Chengdu University of Traditional Chinese Medicine, Chengdu, Sichuan, China; 3 Mianyang Anorectal Hospital, The Eighth People’s Hospital of Mianyang, Mianyang, Sichuan, China

**Keywords:** *Paris polyphylla*, shade-tolerant herb, light adaptation, carbon partitioning, *Paris* saponins, photosynthetic capacity

## Abstract

Shade-tolerant herbs occurring across contrasting radiation environments may differ not only in photosynthetic capacity but also in how assimilated carbon is stored and used. We compared two varieties of *Paris polyphylla* associated with lower- and higher-elevation habitats: *P. polyphylla* var. *chinensis* (PPC) and *P. polyphylla* var. *yunnanensis* (PPY), respectively. Plants were grown for 40 d under light intensity of 50, 200, and 400 μmol m^-^² s^-^¹, and we measured leaf structure, gas exchange, chlorophyll fluorescence, carboxylation traits, non-structural carbohydrates, biomass allocation, and *Paris* saponin VII (PS VII). At 400 μmol m^-^² s^-^¹, PPY had higher net photosynthesis and light-saturated photosynthetic capacity than PPC. Rubisco and Rubisco activase activities, maximum rate of carboxylation and maximum rate of electron transport increased across the irradiance gradient in PPY, even though its stomatal and mesophyll conductance remained lower than in PPC. PPY also maintained a higher relative electron transport rate and a higher photorespiration rate, with little change in non-photochemical quenching (*NPQ*) or dark respiration. Increased irradiance shifted PPY biomass allocation belowground and increased its rhizome/leaf soluble sugar ratio. Foliar PS VII concentration and expression of *PpUGT80A2* and *PpSmt2–1* also increased more strongly in PPY. In PPC, light-saturated photosynthetic capacity peaked at 200 μmol m^-^² s^-^¹, whereas *NPQ* and dark respiration were higher and the leaf soluble sugar/starch ratio was lower at 400 μmol m^-^² s^-^¹. These contrasts indicate that the response of PPY to increased growth irradiance was associated with greater biochemical capacity for CO_2_ assimilation, greater belowground allocation, and a larger foliar saponin pool. Whether saponin synthesis directly alleviates sink limitation or contributes to photoprotection requires direct measurements of carbon flux and oxidative damage.

## 1 Introduction

Light availability varies over short and long spatial scales and is a major determinant of leaf structure, photosynthetic capacity, and whole-plant allocation (Albert et al., 2011; Violle et al., 2012). Shade-tolerant herbs are generally optimized for carbon gain under low irradiance, yet some occur at forest edges or at high elevations where incident radiation is greater and more variable (Valladares and Niinemets, 2008). Under increased irradiance, absorbed energy must be matched by CO_2_ assimilation or safely dissipated; otherwise, the photosynthetic electron transport chain can become over-reduced and the risk of photo-oxidative damage increases (Foyer et al., 2012; Takahashi and Badger, 2011). Comparisons of closely related taxa from contrasting light environments can reveal how these physiological traits vary together. However, responses measured from a limited number of sources in a common environment describe habitat-associated differentiation rather than, by themselves, demonstrating local adaptation or evolutionary canalization (Valladares et al., 2014; Chaves et al., 2026).

Acclimation to growth irradiance involves coordinated changes in light capture, CO_2_ diffusion, and biochemical assimilation. Increased irradiance commonly increases leaf mass per area, alters internal anatomy and chlorophyll investment, and can increase stomatal and mesophyll conductance or nitrogen investment in Rubisco (Evans and Poorter, 2001; Oguchi et al., 2003; Walters, 2005). The relative contribution of each component varies among taxa and environments (Grassi and Magnani, 2005). Greater photosynthetic electron transport must also be balanced by the use or dissipation of excitation energy. Carbon fixation and photorespiration consume ATP and reducing power, whereas non-photochemical quenching (*NPQ*) dissipates excitation as heat; respiration contributes to carbon and energy costs associated with acclimation and maintenance (Takahashi and Badger, 2011; Murchie and Ruban, 2020). These processes are therefore better considered as components of a coordinated energy budget than as mutually exclusive strategies.

Photosynthetic acclimation also depends on the use of carbon after fixation. When export, growth, and storage do not keep pace with assimilation, soluble sugars and starch can accumulate in source leaves and may contribute to feedback regulation of photosynthesis (Paul and Foyer, 2001; Paul and Pellny, 2003). Conversely, allocation to growing or storage organs can maintain sink demand and alter the distribution of non-structural carbohydrates within the plant (Fatichi et al., 2014; Lemoine et al., 2013; Sonnewald and Fernie, 2018). Most studies of irradiance acclimation nevertheless emphasize source capacity, with less attention to whether changes in carbon assimilation are accompanied by changes in organ-level carbon pools. Joint measurements of photosynthesis, biomass allocation, and non-structural carbohydrates can identify such coordination, although static concentrations and biomass ratios do not directly quantify carbon flux.

In highly specialized medicinal plants, complex secondary metabolites, such as steroidal saponins, often account for 2% to 10% or more of total dry biomass (Wink, 2010). This substantial carbon allocation indicates that secondary biosynthesis is not a trace stress-response byproduct, but serves as a substantial destination for primary assimilated carbon (Gershenzon, 1994). Assembling these complex carbon skeletons requires massive investments of energy and reducing power in the form of ATP and NADPH (Selmar and Kleinwächter, 2013). Furthermore, because these synthesized compounds are strictly compartmentalized and stored, rarely re-entering basal metabolic turnover (Yazaki, 2005), they function physiologically as a high-capacity, irreversible carbon sink (Hartmann, 2007). This active metabolic shunting into secondary pathways efficiently consumes excess photoassimilates, directly relieving the photosynthetic feedback inhibition caused by the saturation of primary carbon pools (Paul and Pellny, 2003; Peñuelas and Munné-Bosch, 2005). More importantly, steroidal saponins possess significant non-enzymatic antioxidant activity, allowing their accumulation to directly scavenge intracellular ROS and mitigate lipid peroxidation damage (Selmar and Kleinwächter, 2013). This metabolic strategy, which combines source-sink regulation with non-enzymatic photoprotection, may alleviate light reaction overload under higher light, helping shade-tolerant plants maintain their photosynthetic efficiency.

The genus *Paris L.* consists of shade-tolerant, perennial understory or forest-edge herbs that accumulate substantial amounts of steroidal saponins valued for their antitumor and immunomodulatory pharmacological activities (Ding et al., 2021; eFloras, 2008). Within this phylogeny, *Paris polyphylla* var. *chinensis* (PPC) and *Paris polyphylla* var. *yunnanensis* (PPY) are closely related varieties of *P. polyphylla* that have evolved divergent light-adaptation strategies due to long-term geographic isolation. PPY is endemic to the high-elevation, high-radiation Yunnan-Guizhou Plateau, whereas PPC is widely distributed at low-to-medium elevations within the Yangtze River basin (eFloras, 2008). Prior controlled experiments showed that PPY possesses high-light tolerance, a physiological trait tightly coupled with secondary metabolic flux differentiation; specifically, PPY maintains higher baseline levels of foliar *Paris* saponins (Wen et al., 2023). Recent work also indicates that the secondary metabolic network in *Paris* responds strongly to the light environment, with high light and shortwave ultraviolet radiation specifically inducing the biosynthesis of foliar saponins (Geng et al., 2024). This light-adaptation divergence driven by long-term geographic separation makes *Paris* an ideal model system to resolve how shade-tolerant plants coordinate source-sink relationships to adapt to heterogeneous radiation environments.

In this study, we grew PPC and PPY under three irradiances to systematically evaluate leaf anatomy, gas exchange, chlorophyll fluorescence, Rubisco and Rubisco activase activities, and organ-level carbon allocation. We tested three central hypotheses, (1) high-light adaptation in the PPY relies on the expansion of biochemical carboxylation capacity rather than the optimization of physical CO_2_ diffusion; (2) This upstream biochemical expansion is coordinated with downstream carbon partitioning, specifically through basipetal sugar translocation and foliar steroidal saponin biosynthesis to prevent primary carbohydrate congestion and subsequent photosynthetic feedback inhibition; (3) Alternative biochemical electron sinks, such as photorespiration, operate synergistically with non-photochemical quenching to maintain energy homeostasis and prevent photo-oxidative damage in PPY under severe irradiance. By evaluating responses across anatomical, physiological, and metabolic scales, this research reveals the coordinated physiological mechanisms that allow shade-tolerant herbs to adapt to relatively high-light conditions.

## 2 Materials and methods

### 2.1 Plant materials and experimental design

The 2-year-old plants of PPC and PPY were utilized as experimental materials. The plants were sourced from cultivated bases in Sichuan (30.93°N, 103.55°E, 947 m. a. s. l.) and Yunnan (26.80° N, 100.10° E, 2670 m. a. s. l.), China. Healthy and uniform overwintering rhizomes, devoid of mechanical damage or disease symptoms, were selected for the experiment. Cultivation was conducted in controlled-environment growth chambers located at Chengdu University of Traditional Chinese Medicine (30.68° N, 103.81° E). The growth substrate comprised a homogenized mixture of commercial sphagnum peat moss (Pindstrup, Ryomgaard, Denmark) and vermiculite (3:1, v/v). Individual plants were planted in plastic pots (13 cm diameter × 15 cm depth, one plant per pot).

The experiment was arranged in a randomized complete block design. Plants were subjected to three light intensity treatments: 50, 200, and 400 μmol m^-^² s^-^¹. Each treatment comprised three replicates, with 20 pots per replicate. Irradiance was supplied by red-blue LED arrays (wavelength ranging from 380 to 800 nm; red to blue emission ratio of 3:1). The photosynthetic photon flux density (PPFD) at the canopy level was regularly calibrated using a quantum sensor (PG100N; UPRtek, Taiwan). Environmental conditions within the growth chambers were strictly maintained at a 12-h photoperiod, day/night temperatures of 20 °C/10 °C, and a relative humidity of approximately 85%.

To mitigate localized thermal effects from the light sources, a minimum vertical distance of 50 cm was maintained between the LED arrays and the plant canopy. Pots were arranged in a staggered configuration with a 5-cm spacing to prevent mutual shading, and their positions within the chambers were randomized regularly to eliminate positional artifacts. Following leaf emergence, plants were irrigated weekly with a full-strength Hoagland nutrient solution to ensure non-limiting mineral availability. For all subsequent physiological and biochemical analyses, fully expanded, structurally intact leaves at a uniform developmental stage were selected.

Following 40 days of the respective light treatments, destructive sampling was conducted. Within each biological replicate, 10 representative, healthy plants were randomly selected. Leaves were excised at the petiole insertion point and immediately assessed for leaf area. Plant height was measured with a ruler, and leaf area was analyzed using ImageJ software (National Institutes of Health, Bethesda, MD, USA). Subsequently, the plants were fractionated into stem and belowground compartments. All separated tissues were oven-dried at 70 °C to a constant mass to quantify dry biomass. Dry weights of different organs were recorded. These pooled samples were then ground to a fine, homogeneous powder for subsequent biochemical analyses. Leaf mass per area (LMA= leaf dry mass/leaf area) was detected.

### 2.2 The gas exchange parameters, light response curves, and CO_2_ response curves

Leaf gas exchange parameters were measured using a LI-6800 photosynthesis system (LI-COR Biosciences, USA) at 9:00-11:00 am. The built-in red-blue LED light source was used during measurements. The CO_2_ concentration in the leaf chamber was set to 400 μmol mol^-1^, and photosynthetically active radiation (PAR) was set to 400 μmol m^−2^ s^−1^ (chamber temperature = 20 °C) (Ke et al., 2026). Leaves were fully enclosed in the chamber, and measurements were recorded after stomatal induction and gas exchange parameters had stabilized. Net photosynthetic rate (*P_n_*), stomatal conductance (*g_s_*), intercellular CO_2_ concentration (*C_i_*), and transpiration rate (*T_r_*) were recorded.

*P_n_*-PAR response curves were measured using the automatic program of the LI-6800 system while maintaining the chamber CO_2_ concentration at 400 μmol mol^-1^. Leaves were acclimated in the chamber for 10–20 min before measurements until *P_n_* reached a stable state. The PAR sequence for PPC was 1300, 1100, 900, 800, 600, 400, 200, 150, 100, 50, 20, and 0 μmol m^−2^ s^−1^. The PAR sequence for PPY was 1600, 1400, 1200, 1000, 750, 500, 250, 150, 100, 50, 20, and 0 μmol m^−2^ s^−1^. The resulting data were fitted using a non-rectangular hyperbolic model (Zhang et al., 2020) to estimate apparent quantum yield (*AQY*), maximum net photosynthetic rate (*P_nmax_*), light saturation point (*LSP*), light compensation point (*LCP*), and dark respiration rate (*R_d_*).

*P_n_*-*C_i_* response curves were measured using the LI-6800 system. Leaves were positioned to fully cover the chamber and initially acclimated at a CO_2_ concentration of 400 μmol mol^-^¹. The PAR used for each *P_n_–C_i_* curve was set according to the light-saturation point derived from the corresponding light-response curve, with the treatment-specific values reported together with the relevant results. The CO_2_ concentration sequence was 400, 300, 200, 150, 100, 50, 400, 400, 600, 800, 1000, 1200, 1500, 1800, and 2000 μmol mol^-^¹.

The resulting curves were analyzed in R 4.4.2 (R Core Team, Vienna, Austria) based on the Farquhar-von Caemmerer-Berry model, specifically utilizing the curve-fitting method detailed by Ethier and Livingston (2004). This approach directly incorporates mesophyll conductance (*g_m_*) into the FvCB model, resolving the non-linear relationship between net assimilation rate and intercellular CO_2_ concentration (*C_i_*). From this integrated model, we simultaneously estimated *g_m_*, maximum carboxylation rate (*V_cmax_*), maximum electron transport rate (*J_max_*), maximum net photosynthetic rate (*P_nmax_*), CO_2_ compensation point (*CCP*), CO_2_ saturation point (*CSP*), carboxylation efficiency (*CE*), and photorespiration rate (*R_p_*). Subsequently, true chloroplast CO_2_ concentration (*C_c_*) was calculated as *C_c_= C_i_ - P_n_/g_m_*.

### 2.3 The chlorophyll fluorescence parameters

Before harvest, *in vivo* chlorophyll fluorescence of the leaves was measured using a MINI-PAM-II portable fluorometer (Walz, Effeltrich, Germany). After 30 min of dark adaptation, a 0.6-s saturating pulse (1100 μmol m^-^² s^-^¹) was applied to determine the minimal (*F_0_*) and maximal (*F_m_*) fluorescence. Next, steady-state fluorescence (*F_s_*) and maximum light-adapted fluorescence (*F’_m_*) were measured under continuous actinic light (175 μmol m^-^² s^-^¹ for 60 s). The maximum quantum efficiency of PS II photochemistry (*F_v/_F_m_*), effective quantum yield of PS II [*Y(II)*], photochemical quenching coefficient (*qP*), non-photochemical quenching coefficient (*NPQ*), and relative electron transport rate (*ETR*) were recorded. These parameters were calculated as described previously (Van Kooteen and Snel, 1990; Öquist and Chow, 1992).

Upon completion of the *in vivo* photosynthetic gas exchange and chlorophyll fluorescence measurements, the analyzed leaves and their corresponding rhizomes were rapidly excised, Partial leaf samples were immediately fixed in FAA solution (formalin:acetic acid:ethanol, 1:1:18 v/v/v), the remaining samples were snap-frozen in liquid nitrogen, and stored at −80 °C for biochemical assays.

### 2.4 Leaf nitrogen (N) and carbon (C) concentration

Approximately 10 mg of leaf powder was accurately weighed, wrapped in tin foil, and analyzed using an organic elemental analyzer (Flashsmart, Thermo Fisher Scientific, Waltham, USA) to determine leaf N and C concentration.

### 2.5 The stomatal traits

Stomatal morphology was evaluated utilizing a leaf clearing protocol adapted from Xiong et al. (2018). Vein-free mid-lamina segments (approximately 1 × 0.5 cm) were excised from the FAA-fixed samples. The tissues were sequentially dehydrated through a graded ethanol series (50%, 70%, 85%, 95%, and 100%) for 40 min, and subsequently macerated in 5% (w/v) aqueous NaOH for 48 h. Following two to three rinses with distilled water, the segments were incubated in hydrated trichloroethanol until complete translucency was achieved. The cleared specimens were then stained with a 1% methylene blue solution, rinsed, and mounted on glass slides. Micrographs were acquired utilizing a Nikon Eclipse 50i (Tokyo, Japan) optical microscope, and stomatal parameters, including density and size, were quantified via ImageJ software.

### 2.6 Leaf anatomical structure analysis

Following fixation, the tissue samples were dehydrated through a graded ethanol and n-butanol series, embedded in paraffin wax, and cut into 10-μm-thick transverse sections using a rotary microtome. The sections were double-stained with safranin O and fast green FCF, and permanently mounted in neutral balsam. Anatomical traits were examined and photographed with a Nikon Eclipse 50i light microscope integrated with an ACT-2U digital imaging system (Nikon, Tokyo, Japan). Morphometric extraction, encompassing total leaf thickness and intercellular space area, was executed via Image-Pro Plus software. Subsequently, leaf porosity and mesophyll tissue density were calculated based on established methodologies (Tomeo and Rosenthal, 2017; Théroux-Rancourt and Gilbert, 2017).

### 2.7 The chlorophyll concentration observations

For pigment analysis, frozen leaf samples were ground into a fine powder in liquid nitrogen. Approximately 0.1 g of the powder was extracted in 10 mL of 80% (v/v) acetone at 25 °C for 48 h in the dark (Lichtenthaler and Buschmann, 2001). The absorbance of the supernatant was measured at 645 and 663 nm, and 470 nm using a SpectraMax ID5 spectrophotometer (Molecular Devices, Sunnyvale, CA, USA). Chlorophyll *a* (*Chl a*), chlorophyll *b* (*Chl b*), and carotenoid (*Car*) concentrations were calculated following standard equations (Arnon, 1949; Porra et al., 1989).

### 2.8 Rubisco and Rubisco activase activities

Rubisco activase activity (RCA) was quantified utilizing a plant-specific enzyme-linked immunosorbent assay (ELISA) kit (CB10010-Pt, COIBO BIO Co., Shanghai, China) according to the manufacturer’s protocol. Total Rubisco activity was determined colorimetrically using a commercial Rubisco activity assay kit (BC0445, Solarbio Life Science Co., Beijing, China) (Ke et al., 2026). To prevent thermal denaturation and preserve enzymatic integrity, all extraction and assay procedures were executed strictly at 4 °C.

### 2.9 Determination of soluble sugar (SS) and starch concentration in leaves and rhizomes

Non-structural carbohydrates, encompassing SS and starch, were quantified following the anthrone colorimetric method (Setter and Ella, 1994). For SS extraction, pulverized tissue samples were extracted twice in 80% (v/v) aqueous ethanol at 80 °C for 10 min. The ethanolic supernatants from the dual extractions were pooled for subsequent sugar determination.

To quantify starch, the residual ethanol-insoluble pellet was washed repeatedly with ultrapure water to eliminate residual free sugars. The pellet was then gelatinized in a boiling water bath (100 °C) for 10 min. This gelatinization and extraction procedure was performed twice. The resulting supernatants were pooled, and the solubilized starch was hydrolyzed utilizing perchloric acid. Ultimately, the concentrations of both soluble sugars and starch were determined colorimetrically using the anthrone-sulfuric acid reagent.

### 2.10 Determination of *Paris* saponins in leaves and rhizomes

*Paris* saponin VII (PS VII) represents a predominant steroidal saponin in *Paris* species, constituting a substantial fraction of the total foliar saponin pool (Wen, unpublished). The targeted quantification of PS VII was conducted using an ultra-fast liquid chromatography-tandem mass spectrometry (UFLC-MS/MS) system (Shimadzu, Kyoto, Japan). Comprehensive procedures for sample extraction, chromatographic separation, and subsequent mass spectrometric data acquisition were executed strictly following our previously established protocols (Wen et al., 2023).

### 2.11 Quantitative RT-PCR assays

Total RNA was then extracted using the Total RNA Extractor (Trizol) kit from Sangon Biotech (Shanghai, China) Co., Ltd. RT-PCR was performed using ChamQ Universal SYBR qPCR Master Mix (Vazyme, Nanjing, China). Data were collected from leaf samples of plants grown under 50 and 400 μmol m^-^² s^-^¹, the two light intensities treatment showing the most pronounced phenotypic differences. Expression levels were normalized using *PpACT* as the internal reference gene (Pu et al., 2025). Primers are listed in **Supplementary Table 1**.

### 2.12 Statistical analysis

Statistical analyses were performed using SPSS 25.0 (IBM Corp., Armonk, NY, USA) and R 4.4.2 (R Core Team, Vienna, Austria). For plant height and leaf area, 10 plants were measured within each replicate, whereas three plants per replicate were used for measurements of photosynthetic parameters, chlorophyll fluorescence parameters, stomatal traits, and leaf anatomical traits. Measurements from individual plants within each replicate were averaged, and the replicate mean was used for statistical analyses. Data were first tested for normality and variance homogeneity using the Shapiro-Wilk and Levene’s tests, respectively. Non-compliant data were log-transformed or evaluated with non-parametric alternatives. Outliers were removed only if they resulted from identifiable measurement errors.

A two-way analysis of variance (ANOVA) was used to test the main effects of plant lineage (PPC and PPY), light intensity (50, 200, and 400 μmol m^-^² s^-^¹), and their interaction on the measured physiological and biochemical traits. When ANOVA indicated significant effects, treatment means were compared using Tukey’s honestly significant difference (HSD) test.

Mantel tests were performed in R. Partial least squares structural equation modeling (PLS-SEM) was additionally conducted in SmartPLS 3.0 (SmartPLS GmbH, Boenningstedt, Germany) to explore the multivariate relationships among traits associated with photosynthetic performance and photoprotection. Variety-specific models were fitted separately for PPY and PPC, with nine independent experimental units for each model (three light treatments × three biological replicates; N = 9 per species). Model reliability and validity were confirmed using standard thresholds: Cronbach’s alpha ≥ 0.7, composite reliability ≥ 0.7, and average variance extracted (AVE) ≥ 0.5. Overall model fit was assessed via the goodness-of-fit (GoF) index (Henseler and Sarstedt, 2013), where values > 0.10, 0.25, and 0.36 indicate weak, moderate, and strong explanatory power, respectively (Wetzels et al., 2009). Given the limited sample size, however, the PLS-SEM was treated as an exploratory analysis for summarizing patterns of coordinated trait variation rather than as a confirmatory test of causal relationships.

Figures were created using Origin software (Version 2019; OriginLab Corporation, Northampton, MA, USA) and the R software.

## 3 Results

### 3.1 Effects of light gradients on morphology and nitrogen concentration

**Figure 1:**
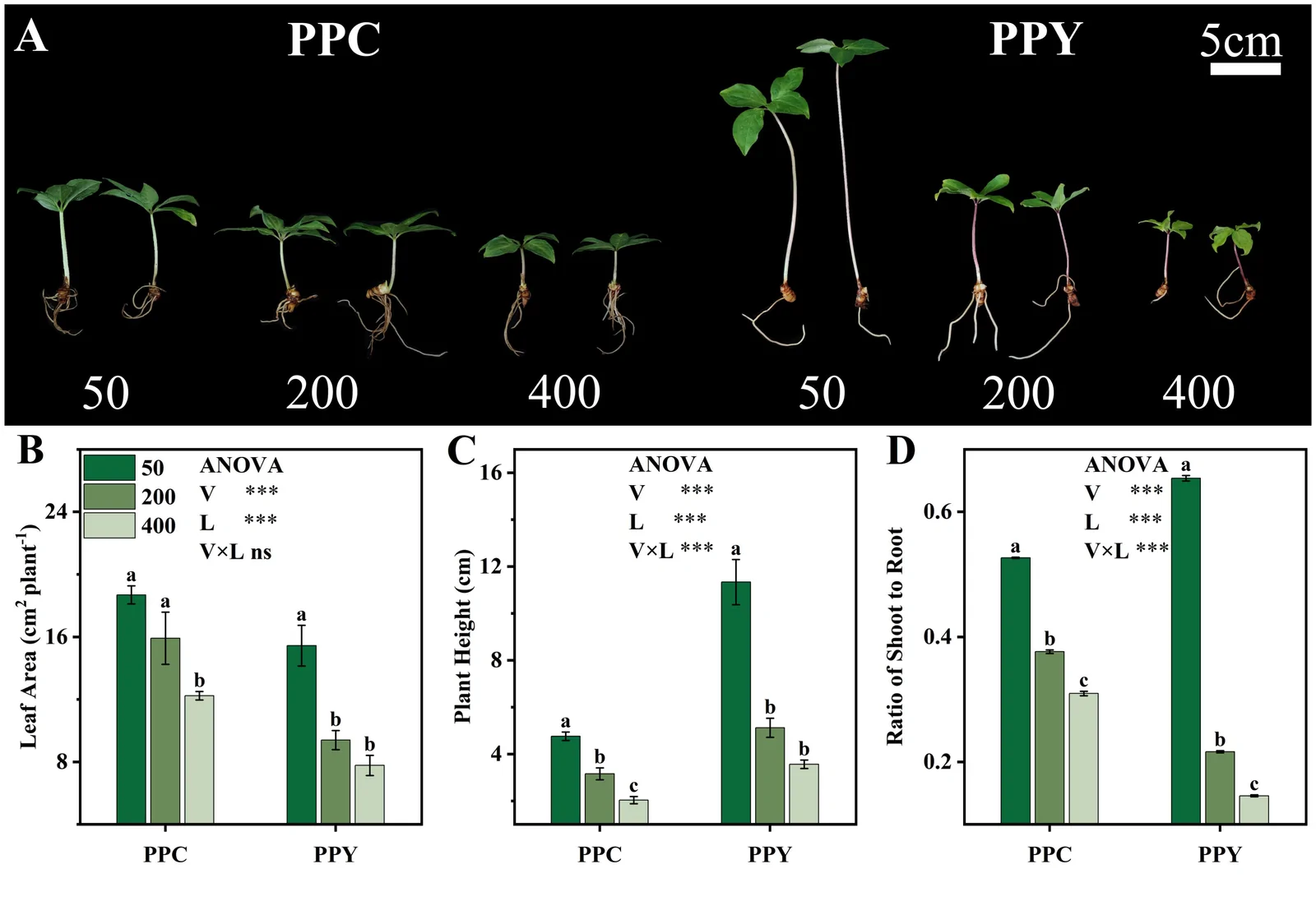
Morphological traits and biomass allocation of *Paris polyphylla* varieties under different light intensities. Whole-plant phenotypes of *P. polyphylla* var. *chinensis* (PPC) and *P. polyphylla* var. *yunnanensis* (PPY) grown under 50, 200, and 400 μmol m^-^² s^-^¹ light intensities **(A)**. Changes in leaf area **(B)**, plant height **(C)**, and ratio of shoot to root dry weight **(D)**. Data are shown as mean ± SE (n=3). Different letters indicate significant differences among treatments (*P* < 0.05). Inset tables summarize two-way ANOVA results for the main effects of variety (V), light (L), and their interaction (V×L) (***P < 0.001, ns, not significant).

Light intensity significantly affected the morphology and biomass allocation of the two *Paris* varieties. Leaf area, plant height, and the shoot/root ratio (S/R) followed a similar trend (**Figure 1**). The parameters decreased as light intensity increased from 50 to 400 μmol m^-^² s^-^¹ (*P* < 0.001). At the lowest light level (50 μmol m^-^² s^-^¹), PPY showed the highest plant height and S/R (**Figures 1C, D**). However, these values dropped sharply in PPY as light increased to 400 μmol m^-^² s^-^¹. In contrast, PPC showed a more stable response across the light gradient. At high light, PPC maintained a higher S/R (0.31) compared to PPY (0.15) (**Figure 1D**).

Leaf N and C concentration also changed with light levels (**Table 1**). Leaf N concentration decreased in both varieties as light increased. In PPC, leaf C concentration increased slightly by 4.51% at higher light. In PPY, leaf C concentration remained stable across all treatments.

**Table 1:** Biomass, photosynthetic and metabolic parameters in PPC and PPY under different light intensities.

Parameters	PPC			PPY		
	50	200	400	50	200	400
Dry weight (g plant ^-1^)						
Belowground	1.63 a^**^	2.37 c	2.34 b	1.19 a	4.14 b^**^	5.78 c^**^
Aboveground	0.86 b^**^	0.89 a	0.72 c	0.77 c	0.90 a	0.84 b^**^
Leaf nitrogen (N) and carbon (C) concentrations (%)						
N	4.37 a^**^	3.86 ab	2.58 b	3.07 a	2.57 ab	2.41 b
C	40.79 b	41.44 b	42.72 a	43.36 a^**^	43.54 a^*^	44.23 a^**^
Gas exchange parameters						
*g_s_* (mol H_2_O m^-2^ s^-1^)	0.20 b^*^	0.20 b	0.33 a^**^	0.06 a	0.12 a	0.11 a
*C_i_* (μmol CO_2_ mol^-1^)	271.87 a	356.56 a	291.63 a	243.74 b	352.40 ab	394.59 a
*T_r_* (mmol H_2_O m^-2^ s^-1^)	1.57 b^*^	2.29 ab	3.21 a^**^	0.35 b	1.04 a	1.32 a
Stomatal morphological traits						
Stomatal size (μm)	0.26 a	0.26 a	0.28 a	0.36 ab^**^	0.34 b^**^	0.40 a^**^
Stomatal aperture area (μm^2^)	0.04 a	0.04 a	0.05 a	0.08 a^**^	0.09 a^*^	0.10 a^**^
Stomatal density (mm^-2^)	31.50 a	34.17 a	33.17 a	22.67 a	32.50 a	36.00 a
Ratios of metabolites						
SS/PS VII	71.96 ab^*^	80.42 a^**^	65.78 b^**^	42.26 a	39.14 a	34.86 a
SS/starch	5.13 a	6.97 a^*^	3.64 a	6.27 a	3.49 b	3.99 b^*^
SS/SS	0.55 a^**^	0.26 b^*^	0.25 b	0.16 b	0.19 ab	0.21 a

The plants of *P. polyphylla* var. *chinensis* (PPC) and *P. polyphylla* var. *yunnanensis* (PPY) grown under 50, 200, and 400 μmol m^-^² s^-^¹ light intensity treatments. Photosynthetic parameters include stomatal conductance (*g_s_*), intercellular CO_2_ concentration (*C_i_*), and transpiration rate (*T_r_*). Metabolic parameters include ratio of leaf soluble sugar to *Paris* saponin VII (SS/PSVII), ratio of leaf soluble sugars to starch (SS/starch), and ratio of rhizome to leaf soluble sugar (SS/SS). Data are presented as mean (n=3). Different letters indicate significant differences among treatments within each variety (*P* < 0.05). * Indicate significant differences between PPY and PPC under the same light intensity treatment (*P < 0.05, **P < 0.01).

### 3.2 Effects of light gradients on photosynthetic characteristics

**Figure 2:**
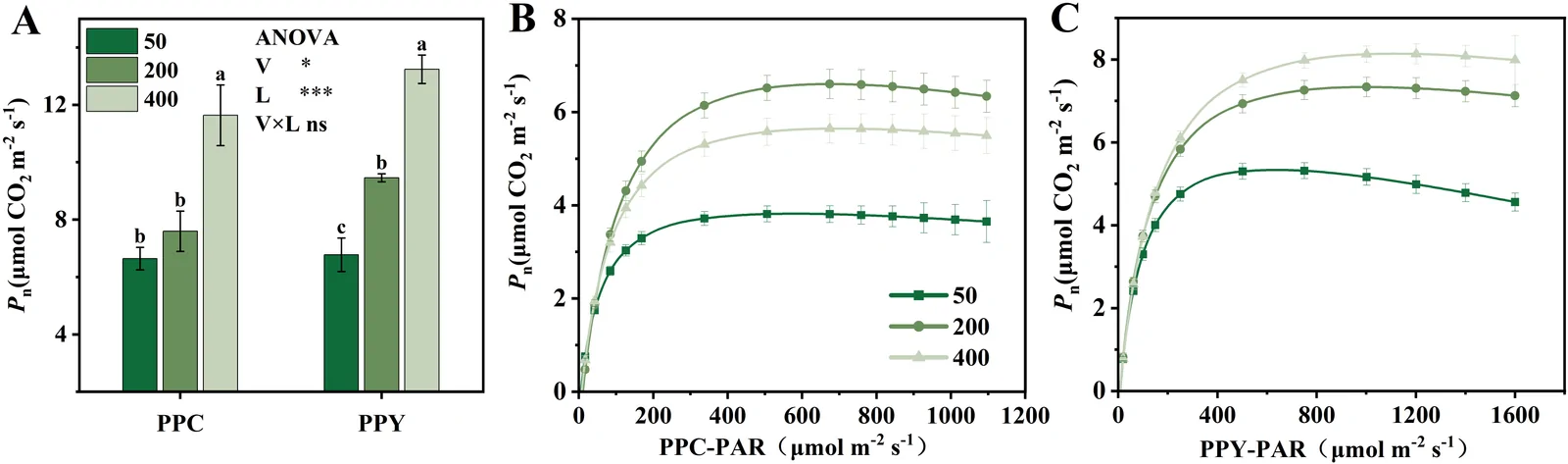
Photosynthetic capacity and light response curves in PPC and PPY. The net photosynthetic rate (*P_n_*) of *P. polyphylla* var. *chinensis* (PPC) and *P. polyphylla* var. *yunnanensis* (PPY) grown under 50, 200, and 400 μmol m^-^² s^-^¹ light intensities **(A)**. Photosynthetic light response curves for PPC **(B)** and PPY **(C)** measured across a range of photosynthetically active radiation (PAR). Data are shown as mean ± SE (n=3). Different letters indicate significant differences among treatments (*P* < 0.05). Inset tables summarize two-way ANOVA results for the main effects of variety (V), light (L), and their interaction (V×L) (*P < 0.05, ***P < 0.001, ns, not significant).

Light intensity significantly affected the photosynthetic performance of the two varieties (**Figure 2**). The *P_n_* increased with higher light treatments in both PPC and PPY (**Figure 2A**). However, the light response curves revealed divergent photosynthetic capacities between the two varieties. For PPC, the maximum *P_n_* were recorded in plants grown under 200 µmol m^-2^ s^-1^ (**Figure 2B**). In contrast, PPY showed a continuous increase in *P_n_* across the light gradient (**Figure 2C**). At 400 μmol m^-^² s^-^¹, the *P_n_* of PPY was significantly higher than that of PPC. The light saturation point showed a similar variety-specific response. Under high light (400 μmol m^-^² s^-^¹), the *I_sat_* of PPY increased significantly to 1143.6 μmol m^-^² s^-^¹ (**Supplementary Table 2**). At the same light level, PPC maintained a much lower *I_sat_*.

Leaf photosynthetic pigments and other gas exchange parameters also varied significantly between the treatments (**Supplementary Table 3**; **Table 1**). For leaf pigments, the total chlorophyll concentration in PPY decreased steadily as light intensity increased. In PPC, total chlorophyll peaked at 200 μmol m^-^² s^-^¹ and then decreased under 400 μmol m^-^² s^-^¹. In both varieties, the chlorophyll *a*/*b* ratio increased with light intensity, whereas no significant difference was detected between the 200 and 400 µmol m^-^² s^-^¹ (**Supplementary Table 3**). For gas exchange parameters, *g_s_* differed remarkably between the two varieties. Across all light treatments, the *g_s_* of PPY was significantly lower than that of PPC (**Table 1**). Furthermore, the *R_d_* showed clear variety-specific patterns (**Supplementary Table 2**). The *R_d_* of PPC increased alongside light intensity, whereas the *R_d_* of PPY showed no significant variation across the light gradient.

### 3.3 Effects of light gradients on chlorophyll fluorescence

**Figure 3:**
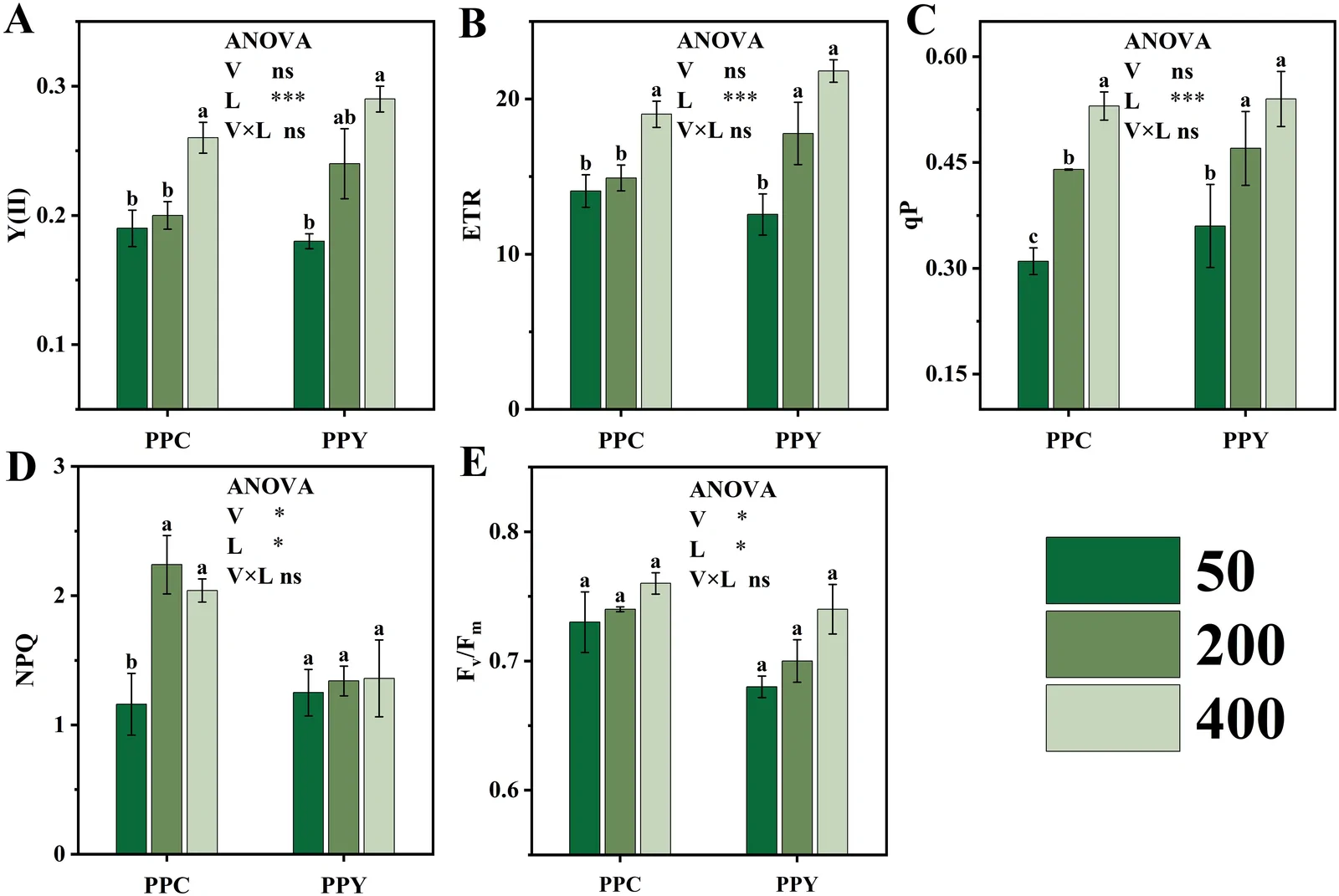
Chlorophyll fluorescence parameters in response to varying light intensities. Effective quantum yield of PS II (*Y(II)*) **(A)**, electron transport rate (*ETR*) **(B)**, photochemical quenching (*qP*) **(C)**, non-photochemical quenching (*NPQ*) **(D)**, and maximum quantum yield of PSII (*F_v_*/*F_m_*) **(E)** in *P. polyphylla* var. *chinensis* (PPC) and *P. polyphylla* var. *yunnanensis* (PPY) under 50, 200, and 400 μmol m^-^² s^-^¹ light treatments. Data are shown as mean ± SE (n=3). Different letters indicate significant differences among treatments (*P* < 0.05). Inset tables summarize two-way ANOVA results for the main effects of variety (V), light (L), and their interaction (V×L) (*P < 0.05, ***P < 0.001, ns, not significant).

Light intensity significantly altered the chlorophyll fluorescence parameters of both varieties (**Figure 3**). The *Y(II)*, *ETR*, and *qP* increased with higher light treatments in both PPC and PPY (*P* < 0.001) (**Figures 3A–C**). However, the two varieties exhibited different response magnitudes. Under the highest light treatment (400 μmol m^-^² s^-^¹), PPY achieved higher *Y(II)* and *ETR* compared to PPC (**Figures 3A, B**). Notably, the *ETR* of PPY showed a steeper increase across the light gradient, reaching its maximum value at 400 μmol m^-^² s^-^¹.

The most remarkable variety-specific difference occurred in *NPQ* (**Figure 3D**). In PPC, *NPQ* increased sharply when light intensity rose from 50 to 200 μmol m^-^² s^-^¹, and it remained at a high level under 400 μmol m^-^² s^-^¹. Conversely, the *NPQ* of PPY remained relatively low and stable across all light treatments, showing no significant variation. Regarding the *F_v_*/*F_m_*, neither variety exhibited significant fluctuations across the light gradient (**Figure 3E**).

### 3.4 Effects of light gradients on CO_2_ assimilation

**Figure 4:**
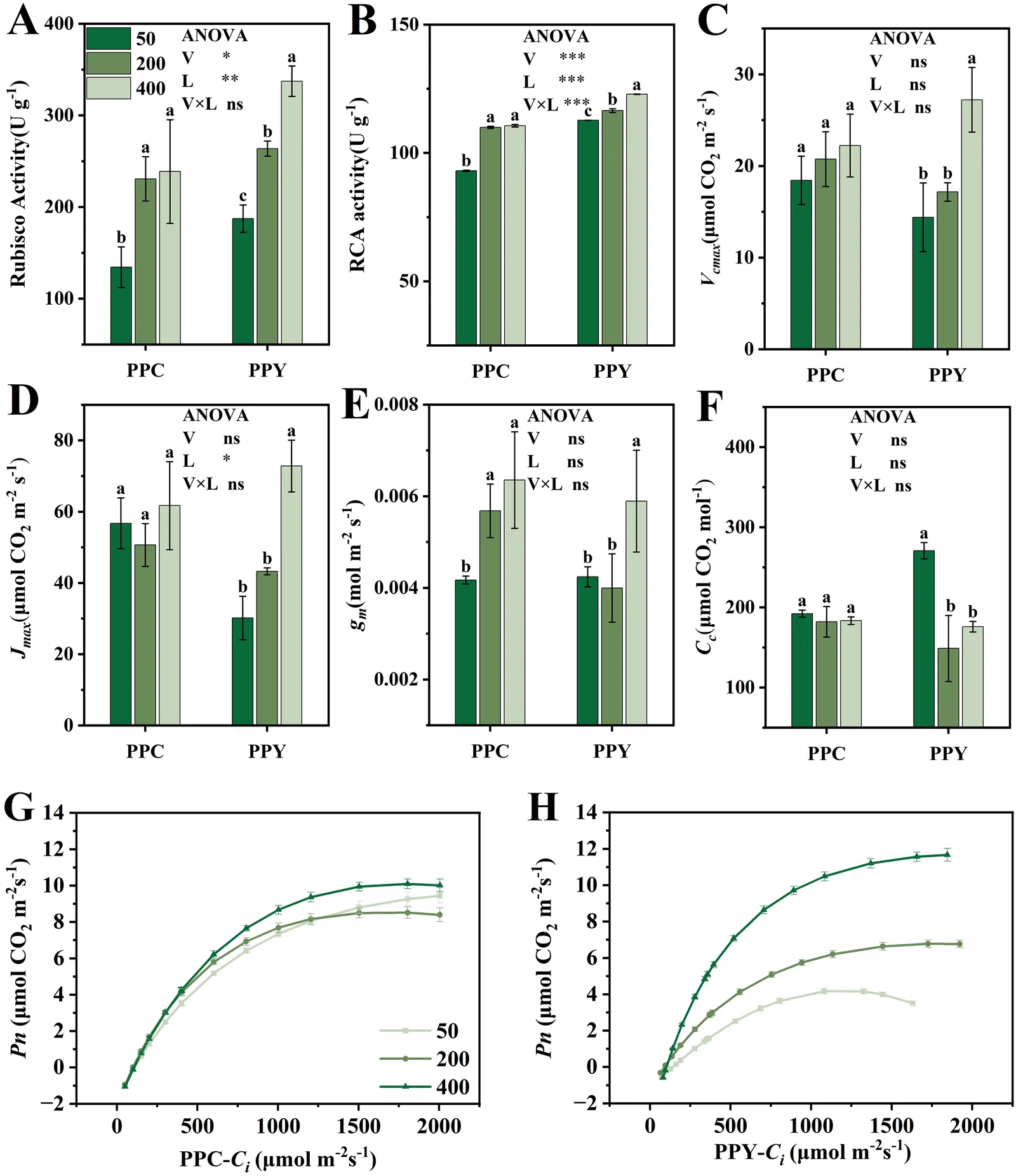
Biochemical carbon fixation capacity and diffusion conductance in PPC and PPY. Rubisco activity **(A)**, and Rubisco activase activity (RCA) **(B)**, Maximum rate of carboxylation (*V_cmax_*) **(C)**, maximum rate of electron transport (*J_max_*) **(D)**, mesophyll conductance (*g_m_*) **(E)**, chloroplast CO_2_ concentration (*C_c_*) **(F)**, net photosynthetic rate (*P_n_*) in response to varying intercellular CO_2_ concentrations (*C_i_*) for *P. polyphylla* var. *chinensis* (PPC) **(G)** and *P. polyphylla* var. *yunnanensis* (PPY) **(H)** grown under 50, 200, and 400 μmol m^-^² s^-^¹ light treatments. Data are shown as mean ± SE (n=3). Different letters indicate significant differences among treatments (*P* < 0.05). Inset tables summarize two-way ANOVA results for the main effects of variety (V), light (L), and their interaction (V×L) (*P < 0.05, **P < 0.01, ***P < 0.001, ns, not significant).

Light intensity significantly enhanced the biochemical capacity for CO_2_ assimilation in both varieties (**Figure 4**). However, PPC and PPY exhibited divergent response patterns in the activities of Rubisco and RCA (**Figures 4A, B**). In PPY, Rubisco and RCA activities increased continuously across the light gradient, reaching their maximum values at 400 μmol m^-^² s^-^¹. In contrast, the activities of these enzymes in PPC increased from 50 to 200 μmol m^-^² s^-^¹ and then remained stable under 400 μmol m^-^² s^-^¹. Both *V_cmax_* and the *J_max_* also showed clear variety-specific differences (**Figures 4C, D**). In PPC, both *V_cmax_* and *J_max_* remained stable across all light treatments. Conversely, in PPY, these parameters increased with light intensity and peaked at 400 μmol m^-^² s^-^¹.

The *g_m_* and *C_c_* also showed variety-specific responses (**Figures 4E, F**). The *g_m_*of PPY increased significantly at 400 μmol m^-^² s^-^¹, whereas PPC showed no significant difference between the 200 and 400 µmol m^-^² s^-^¹ (**Figure 4E**). However, *C_c_* revealed a contrasting pattern. In PPC, *C_c_* remained stable across all light treatments with no significant differences (**Figure 4F**). In contrast, the *C_c_* of PPY decreased significantly under 200 and 400 μmol m^-^² s^-^¹ compared to the low light treatment (**Figure 4F**).

The CO_2_ response curves and fitted parameters further demonstrated divergent assimilation strategies (**Figure 4**; **Supplementary Table 4**). For the *CCP*, PPC maintained stable values across the light gradient (**Supplementary Table 4**). Conversely, the *CCP* of PPY increased sharply to 163.74 μmol mol^-1^ under 400 μmol m^-^² s^-^¹. Furthermore, the *R_p_* exhibited a similar contrasting trend (**Supplementary Table 4**). Under high light conditions, the *R_p_* of PPY increased dramatically by approximately 3-fold. In comparison, the *R_p_* of PPC showed no difference between 200 and 400 μmol m^-^² s^-^¹.

### 3.5 Effects of light gradients on leaf anatomical structures

**Figure 5:**
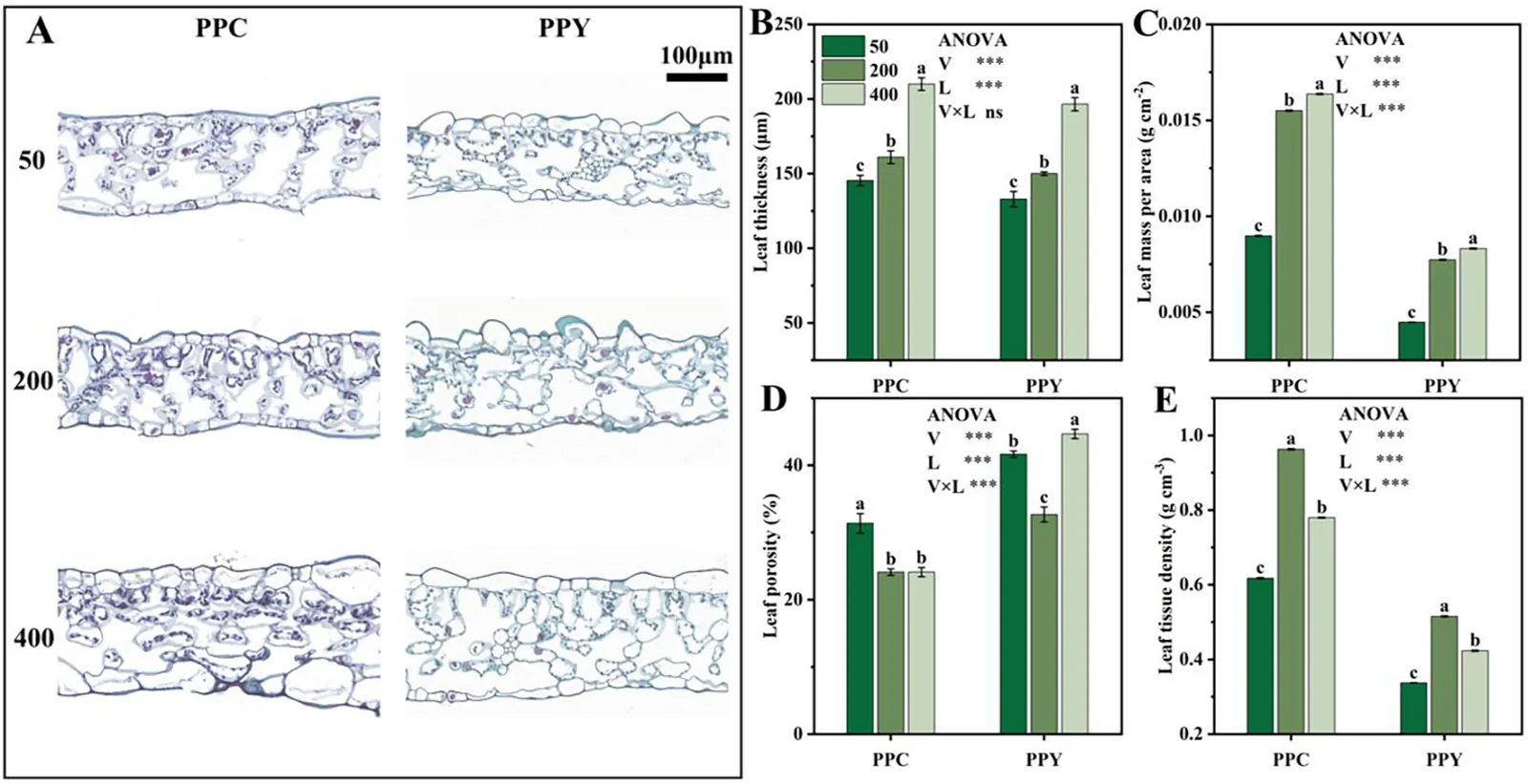
Leaf anatomical traits of PPC and PPY under different light intensities. The plants of *P. polyphylla* var. *chinensis* (PPC) and *P. polyphylla* var. *yunnanensis* (PPY) grown under 50, 200, and 400 μmol m^-^² s^-^¹ light intensities. Representative light micrographs of leaf cross-sections from PPC and PPY **(A)**. Quantitative anatomical changes in leaf thickness **(B)**, leaf mass per area **(C)**, leaf porosity **(D)**, and leaf tissue density **(E)**. Data are shown as mean ± SE (n=3). Different letters indicate significant differences among treatments (*P* < 0.05). Inset tables summarize two-way ANOVA results for the main effects of variety (V), light (L), and their interaction (V×L) (***P < 0.001, ns, not significant).

Light intensity significantly altered the leaf anatomical structures of both varieties (**Figure 5**). Leaf thickness increased continuously with higher light treatments in both PPC and PPY (*P* < 0.001) (**Figures 5A, B**). However, LMA showed a highly significant interaction between variety and light (V × L, *P* < 0.001). Under the 400 μmol m^-^² s^-^¹, the LMA of PPC increased sharply to its maximum value, which was significantly higher than that of PPY under the same light condition (**Figure 5C**). The most striking variety-specific divergence was observed in leaf porosity (**Figure 5D**) and leaf tissue density (**Figure 5E**). In PPC, leaf porosity decreased significantly from 50 to 200 μmol m^-^² s^-^¹ and remained at a low level under 400 μmol m^-^² s^-^¹. Conversely, the leaf porosity of PPY reached its highest value under the 400 μmol m^-^² s^-^¹ light treatment. Consistent with this structural divergence, the leaf tissue density of PPC was consistently and significantly higher than that of PPY across all light treatments.

Stomatal traits also exhibited variety-specific differences (**Table 1**). Across the light gradient, the stomatal size and stomatal aperture area of PPY were consistently larger than those of PPC. However, stomatal density did not show significant variations across the treatments for either variety. Furthermore, light intensity had no significant impact on stomatal aperture area or stomatal density in either variety.

### 3.6 Effects of light gradients on carbohydrate metabolism and secondary metabolite accumulation

**Figure 6:**
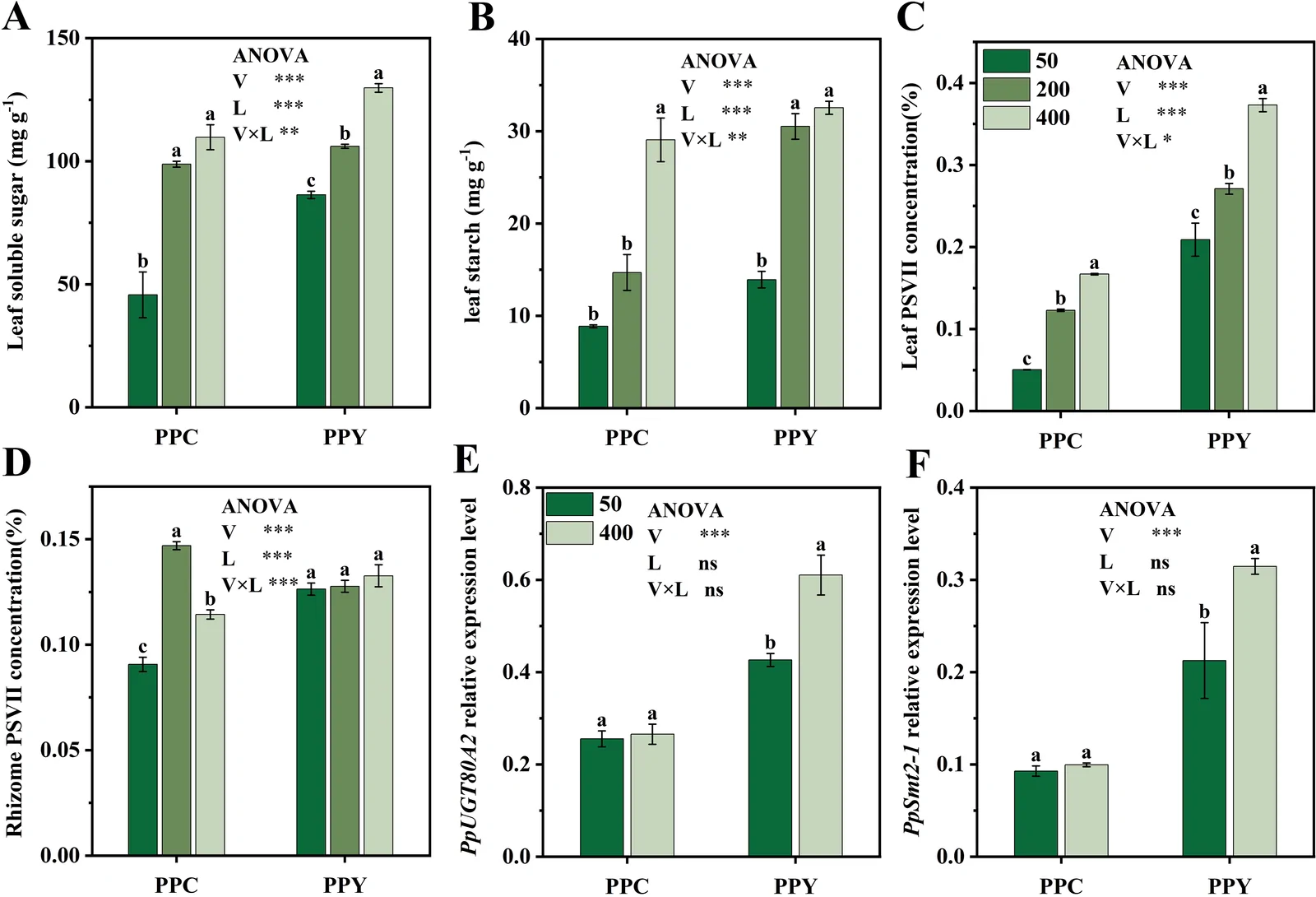
The concentration of non-structural carbohydrates and PS VII, and expression of related biosynthetic genes in PPC and PPY. The concentrations of leaf soluble sugar **(A)**, leaf starch **(B)**, leaf *Paris* saponin VII (PS VII) **(C)**, and rhizome PS VII **(D)** in *P. polyphylla* var. *chinensis* (PPC) and *P. polyphylla* var. *yunnanensis* (PPY) grown under 50, 200, and 400 μmol m^-^² s^-^¹ light treatments. Relative expression levels of key secondary metabolic genes *UGT80A2* **(E)** and *Smt2-1* **(F)**. Data are shown as mean ± SE (n=3). Different letters indicate significant differences among treatments (*P* < 0.05). Inset tables summarize two-way ANOVA results for the main effects of variety (V), light (L), and their interaction (V×L) (*P < 0.05, **P < 0.01, ***P < 0.001, ns, not significant).

Light intensity significantly altered the carbohydrate metabolism and utilization strategies of the two varieties (**Table 1**; **Figure 6**). The absolute concentration of non-structural carbohydrates (SS and starch) in leaves and rhizomes generally increased under high light for both varieties (**Figures 6A, B**). In PPC, the leaf SS/Starch ratio peaked at 200 μmol m^-^² s^-^¹ and decreased at 400 μmol m^-^² s^-^¹ (**Table 1**). In contrast, the leaf SS/Starch ratio of PPY decreased under higher light treatments. The spatial partitioning of SS, represented by the rhizome to leaf SS ratio (SS/SS), also diverged (**Table 1**). PPY maintained a significantly lower SS/SS than PPC across all light treatments. However, as light intensity increased, the SS/SS of PPC decreased, whereas the SS/SS of PPY increased steadily.

Regarding secondary metabolism, the ratio of SS to PS VII in leaf was significantly lower in PPY than in PPC across all light intensities (**Table 1**). This ratio decreased under high light in both varieties. Correspondingly, the concentration of leaf PS VII increased significantly under high light, with PPY accumulating much higher levels than PPC (**Figure 6C**). The relative expression levels of the regulatory genes *PpUGT80A2* and *PpSmt2–1* were higher under 400 than under 50 μmol m^-^² s^-^¹ treatment. Notably, PPY showed a more pronounced up-regulation of these genes compared to PPC (**Figures 6E, F**).

**Figure 7:**
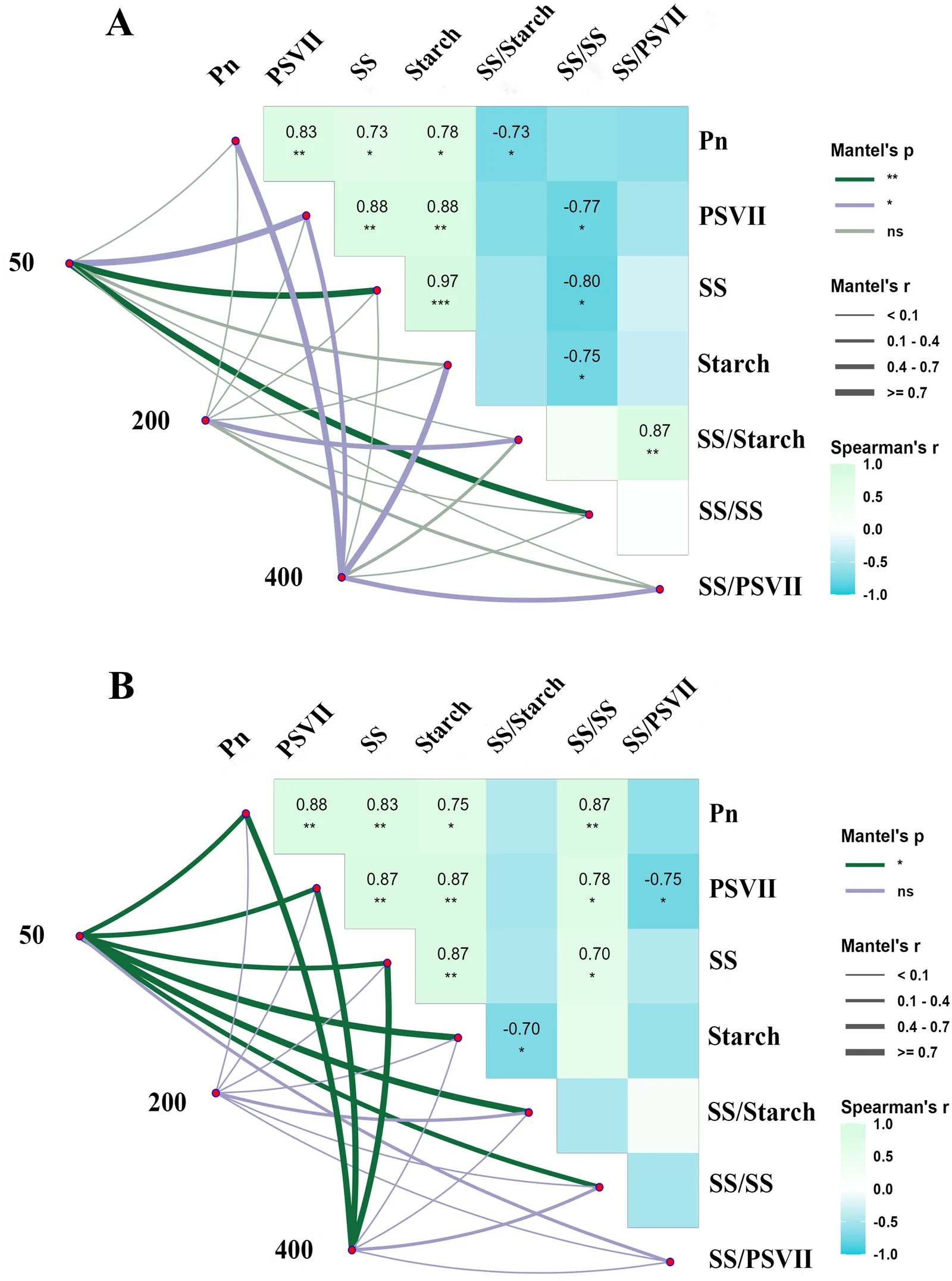
Correlation networks among photosynthetic performance, primary carbohydrates, and secondary metabolites. Mantel test and Spearman’s correlation analysis for *P. polyphylla* var. *chinensis* (PPC) **(A)** and *P. polyphylla* var. *yunnanensis* (PPY) **(B)** across different light intensities (50, 200, and 400 μmol m^-^² s^-^¹). Edge width corresponds to the Mantel’s r statistic, and edge colour indicates statistical significance (Mantel’s p). The heatmap displays Spearman’s correlation coefficients among net photosynthetic rate (*P_n_*), concentration of leaf *Paris* saponin VII (PS VII), soluble sugar (SS), starch, and their respective ratios (SS/Starch, SS/PS VII). SS/SS, ratio of rhizome soluble sugar to leaf soluble sugar. (*P < 0.05, **P < 0.01, ***P < 0.001, ns, not significant).

The Mantel test and correlation networks further revealed distinct systemic integration between primary and secondary metabolism in the two varieties (**Figure 7**). In both varieties, light intensity exhibited strong positive correlations with the *P_n_*, PS VII, SS, starch, and SS/SS. However, the correlation network topologies diverged fundamentally in the metabolic partitioning ratios. In PPC, *P_n_* was significantly negatively correlated with the leaf SS/Starch ratio, and PS VII displayed a significant negative correlation with SS/SS (**Figure 7A**). Conversely, both *P_n_* and PS VII exhibited strong significant positive correlations with SS/SS in PPY (**Figure 7B**). Such contrasting association patterns reveal profound variety-specific trade-offs driven by the spatial partitioning of sugars.

### 3.7 Structural relationships among photosynthetic traits and metabolic partitioning

**Figure 8:**
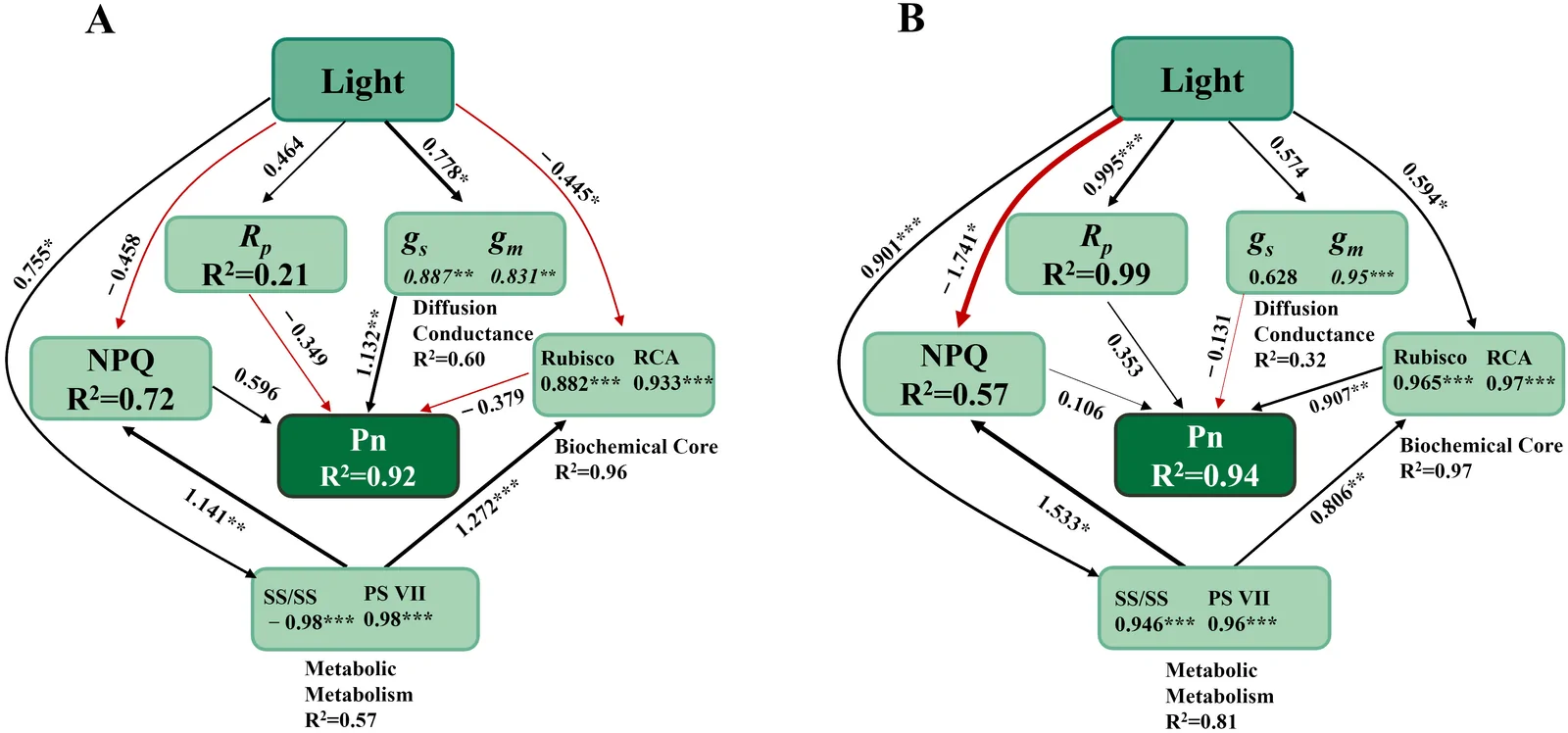
Structural equation models (SEM) delineating the synergy among light reactions, dark reactions, and carbon metabolism. SEM models illustrating the direct and indirect effects of light intensity on photoprotection, diffusion conductance, biochemical core capacity, net photosynthetic rate, and downstream carbon metabolism in *P. polyphylla* var. *chinensis* (PPC) **(A)** and *P. polyphylla* var. *yunnanensis* (PPY) **(B)**. Solid black and red arrows represent significant positive and negative pathways, respectively. Arrow thickness is proportional to the path coefficient. The proportion of variance explained (R²) is shown for each endogenous variable. *R_p_*, photorespiration rate; *NPQ*, non-photochemical quenching; *g_s_*, stomatal conductance; *g_m_*, mesophyll conductance; *P_n_*, net photosynthetic rate; RCA, Rubisco activase activity; SS/SS, ratio of rhizome soluble sugar to leaf soluble sugar. (*P < 0.05, **P < 0.01, ***P < 0.001).

PLS-SEM was used as an exploratory approach to evaluate the relationships among growth irradiance, photorespiration, diffusional conductance, biochemical capacity, *NPQ*, metabolic partitioning, and *P_n_* in PPC and PPY (**Figure 8**). The models showed high explained variance for *P_n_* in PPC and PPY (R² = 0.92 and 0.94, respectively). In PPC, irradiance was positively associated with diffusional conductance, which showed a positive relationship with *P_n_*, whereas biochemical capacity exhibited a weaker contribution to *P_n_* (**Figure 8A**). In contrast, PPY showed stronger coordination between biochemical capacity and photosynthetic performance, with biochemical capacity showing a positive association with *P_n_*, while the contributions of *R_p_* and diffusional conductance were relatively limited (**Figure 8B**).

Irradiance was positively associated with metabolic partitioning in both PPC and PPY, explaining 57% and 81% of its variation, respectively. Metabolic partitioning was positively associated with *NPQ* and biochemical capacity in both lineages. In PPY, irradiance showed a negative association with *NPQ*, suggesting a shift in photoprotective regulation under high irradiance.

## 4 Discussion

### 4.1 Contrasting morphological plasticity and biomass allocation

Spatiotemporal light heterogeneity drives plant phenotypic and resource allocation divergence. According to the leaf economics spectrum and optimal partitioning theory, light-adapted plants exhibit high plasticity and acquisitive traits, whereas shade-tolerant plants maintain stable morphologies and conservative strategies to minimize maintenance costs (Wright et al., 2004). Here, the contrasting phenotypic responses of PPY and PPC to the light gradient highlight their geographically isolated adaptive strategies (**Figure 1**).

PPY exhibited massive plasticity in leaf area and plant height (**Figure 1**). Crucially, its shoot to root biomass ratio decreased significantly under 400 μmol m^-^² s^-^¹, reflecting a strategic shift towards belowground investment (**Figure 1D**). This flexible inter-organ resource reallocation characterizes a pronounced resource-acquisitive strategy (Poorter et al., 2012; Freschet et al., 2018). Conversely, PPC maintained stable morphology and biomass allocation across treatments (**Figure 1**), reflecting a conservative strategy adapted to shaded habitats to circumvent high construction costs (Valladares and Niinemets, 2008).

While LMA increased with irradiance in both varieties (**Figure 5C**), reflecting a typical anatomical response to high irradiance (Niinemets and Keenan, 2014), PPY consistently exhibited lower LMA and larger mesophyll intercellular spaces than PPC (**Figures 5C–E**), despite similar leaf thicknesses (**Figure 5B**). This is thick but low-density structure minimizes dry matter construction costs (John et al., 2017). More importantly, this high porosity is a critical optimization against intense radiation. In overly dense leaves, adaxial mesophyll cells face photoinhibition while deeper cells suffer light starvation (Terashima et al., 2009). PPY’s large intercellular spaces increase the optical path length, inducing strong internal light scattering. This “light homogenization” mitigates adaxial photodamage and distributes energy evenly, enhancing whole-leaf light-use efficiency (Vogelmann, 1993).

Under low light, PPC exhibited higher leaf nitrogen (**Table 1**) and chlorophyll concentrations (**Supplementary Table 3**), typical of shade-tolerant plants investing heavily in light-harvesting complexes (Evans and Poorter, 2001). With increasing light, these concentrations declined in both lineages, likely due to a structural dilution effect driven by non-structural carbohydrate accumulation (Ke et al., 2023; Wen et al., 2023). Furthermore, optimal nitrogen partitioning dictates that this chlorophyll reduction reflects an adaptive downsizing of nitrogen-costly antennae, reallocating nitrogen toward downstream assimilation enzymes (Niinemets, 2007). The significantly up-regulated Rubisco activity in PPY under 400 μmol m^-^² s^-^¹ corroborates this strategic shift from light interception to biochemical carbon fixation (**Figure 4A**). Overall, PPY’s lower construction costs and highly plastic structure provide the necessary morphological foundation to sustain high metabolic fluxes under severe irradiance.

### 4.2 Biochemical and diffusive components of photosynthetic acclimation

Photosynthetic carbon assimilation is fundamentally constrained by the interplay between CO_2_ diffusion and biochemical carboxylation fluxes. While high mesophyll porosity typically facilitates gas-phase transport (Flexas et al., 2010), environmental stressors often induce a decoupling between leaf anatomical limits and actual diffusive capacity (Flexas and Carriquí, 2020). Under such conditions, the maintenance of photosynthetic flux becomes heavily reliant on the operational efficiency of the carbon-fixing enzymatic system (Grassi and Magnani, 2005). In this study, PPY and PPC exhibited distinct gas exchange and biochemical strategies in response to high light.

PPC maintained relatively *g_s_* and *g_m_* conductance across light treatments (**Table 1**; **Figure 4E**), with chloroplast *C_c_* remaining stable (**Figure 4F**). This suggests that the shade-tolerant PPC remains dependent on maintaining open physical diffusion pathways for carbon supply. However, these elevated conductance levels did not translate into significant photosynthetic gains (**Figure 2**); the constant *C_c_* reflects a static equilibrium between supply and consumption, indicating that photosynthetic potential in PPC is primarily limited by its lower biochemical carboxylation capacity (**Figure 4**) (Grassi and Magnani, 2005). In contrast, PPY displayed atypical gas exchange. Despite its highly porous mesophyll, PPY exhibited lower *g_s_* and *g_m_* than PPC (**Table 1**; **Figure 4E**), suggesting that its acclimation to high light was not associated with an enhancement of CO_2_ diffusional capacity. Instead, RCA and Rubisco activities increased markedly under higher irradiance (**Figures 4A, B**), indicating a stronger biochemical adjustment of the carboxylation system. Consistent with these treatment responses, the exploratory PLS-SEM showed a positive association between Rubisco activity and *Pn* in PPY (**Figure 8**). Notably, *C_c_* declined markedly under high light (**Figure 4F**), consistent with biochemical CO_2_ consumption increasing relative to diffusional replenishment at the carboxylation sites (Yamori et al., 2012).

This low conductance-high biochemical consumption model in PPY reflects a strategic trade-off between carbon fixation and water conservation. In high-altitude habitats like the Yunnan-Guizhou Plateau, intense radiation is frequently coupled with high vapor pressure deficit. Despite possessing larger stomatal anatomical potential, PPY maintained lower *g_s_* and *T_r_* than PPC (**Table 1**), mitigating the risk of excessive water loss under severe irradiance (Brodribb and McAdam, 2017). The physiological foundation for bypassing high physical conductance lies in PPY’s highly plastic Rubisco capacity (**Figure 4A**), which compensates for the reduced CO_2_ supply. By bypassing structural conductance limitations, PPY optimizes intrinsic water-use efficiency while sustaining high carbon assimilation (Cowan and Farquhar, 1977), an adaptation well-suited to the high-radiation conditions of high-altitude environments.

### 4.3 Contrasting energy-use and dissipation responses

Under high-irradiance stress, maintaining energy homeostasis in photosystems requires the synergistic coordination of multiple energy dissipation pathways. When absorbed light energy exceeds the biochemical consumption capacity of carbon fixation, plants protect the photosynthetic electron transport chain either through *NPQ*-mediated thermal dissipation or by relying on alternative electron sinks, such as photorespiration, to avert the accumulation of reactive oxygen species and subsequent oxidative damage (Takahashi and Badger, 2011; Alric and Johnson, 2017; Murchie and Ruban, 2020). In this study, the two *Paris* varieties exhibited adaptive divergence in their energy dissipation and photoprotective strategies when exposed to high radiation.

The PPC relied heavily on passive thermal dissipation. Under 400 μmol m^-^² s^-^¹, PPC maintained significantly higher *NPQ* levels than PPY, which continuously intensified with increasing irradiance (**Figure 3D**). However, its *P_nmax_* peaked at 200 μmol m^-^² s^-^¹ and remained significantly lower than that of PPY (**Supplementary Table 2**). Furthermore, the *R_d_* of PPC surged drastically with increasing light intensity (from 0.41 to 0.87) (**Supplementary Table 2**). This extreme plasticity in dark respiration is an evolutionary hallmark of shade-tolerant plants. In low-radiation environments, they maintain minimal basal respiration to conserve energy; yet, when exposed to high-light stress, they possess the physiological machinery to rapidly up-regulate respiratory metabolic fluxes to fuel damage repair (Valladares and Niinemets, 2008). This process consumes substantial amounts of primary assimilates to sustain the degradation and *de novo* synthesis turnover of core photosynthetic proteins (e.g., the D1 protein in the PS II reaction center) (Tcherkez et al., 2017; O'Leary et al., 2019). While this strategy of passive thermal dissipation and costly repair suits episodic understory sunflecks, it severely compromises net carbon gain under sustained high light. The exponentially increased maintenance costs ultimately constitute a profound physiological limitation for PPC under chronic high light.

Conversely, PPY deployed a photoprotective strategy centered on biochemical dissipation. Under high light, PPY sustained a high *ETR*, driving the robust formation of assimilatory power (ATP and NADPH) (**Figure 3B**). Under severe irradiance, PPY engaged multiple energy diversion pathways. A substantial portion of this assimilatory power was directly utilized by its robust carbon fixation engine (**Figure 4**); simultaneously, the excess assimilatory power was effectively dissipated by up-regulating the *R_p_*(**Supplementary Table 4**). As a critical alternative biochemical sink under severe irradiance, the active *R_p_* pathway efficiently consumes excess reducing equivalents, ensuring the smooth operation of the electron transport chain and preventing over-reduction (Kozaki and Takeba, 1996; Busch, 2020). Benefiting from this high carbon fixation coupled with photorespiratory energy relief strategy, the photosynthetic apparatus of PPY effectively evaded high-light-induced oxidative damage. This is further corroborated by its consistently low and stable *R_d_* across the entire light gradient (**Supplementary Table 2**), indicating minimal repair costs. Ultimately, supported by these efficient photoprotective mechanisms, PPY demonstrated a notably broader ecological light niche: it exhibited not only a lower *I_c_* than PPC but also a substantially higher *I_sat_* (**Supplementary Table 2**), thereby sustaining a superior maximum net photosynthetic rate.

### 4.4 Biomass allocation, carbohydrate pools, and saponin accumulation

In high-radiation habitats, efficient cross-organ translocation and *in situ* utilization of photosynthates are prerequisites for sustaining photosynthetic efficiency (Paul and Foyer, 2001; Sonnewald and Fernie, 2018). Here, the two *Paris* varieties exhibited divergence in carbon partitioning under high-light stress. PPY demonstrated highly efficient basipetal assimilate flux and foliar secondary metabolic diversion, facilitating its adaptation to the high-radiation plateau.

Under severe irradiance, PPY exhibited enhanced basipetal transport, marked by a light-dependent decreases in the aboveground to belowground dry matter ratio (**Figure 1D**). This preferential carbon partitioning to roots aligns with typical sun-adapted plants (Poorter et al., 2012; Lemoine et al., 2013; Zhou et al., 2019, 2020, 2021). In contrast, this ratio decreased in the shade-tolerant PPC (**Figure 1D**), indicating a severe bottleneck in assimilate export. Consequently, massive photosynthates were retained in PPC source leaves and converted into starch, significantly reducing its sugar/starch ratio at 400 μmol m^-^² s^-^¹ (**Table 1**; **Figure 6**). The positive correlation between *P_n_* and the rhizome-to-leaf soluble sugar ratio (**Figure 7**) is consistent with a potential role of cross-organ carbon export in maintaining photosynthetic performance. Excessive foliar sugar triggers signaling-mediated feedback inhibition (Paul and Eastmond, 2020), identifying *in situ* carbon congestion as the primary driver of the severe *P_n_* decline in PPC under sustained high light.

Beyond cross-organ export, PPY employs a high-throughput secondary metabolic shunt within leaves to further circumvent feedback inhibition. Our previous research showed that PS VII is strongly induced by high light and primarily synthesized in leaves (Wen et al., 2023). High irradiance drives the trans-cellular transport and spatial compartmentalization of these saponins into epidermal cells (Wen et al., 2023). This expands storage capacity while protecting the mesophyll photosynthetic apparatus from secondary metabolite toxicity. Consistent with this, foliar PS VII in PPY exhibited robust light-dependent accumulation (**Figure 6C**), mirroring the up-regulation of key biosynthetic genes (*PpUGT80A2* and *PpSmt2-1*) and exceeding PPC levels (**Figure 6E, F**). Notably, the soluble sugar to PS VII ratio in PPY dropped drastically to 34.86 at 400 μmol m^-^² s^-^¹ (versus 65.78 in PPC) (**Table 1**). Supporting the overflow metabolism hypothesis, PPY utilizes the *Paris* saponin pathway as an alternative biochemical sink, drawing excess sugars away from the primary pool to unlock the photosynthetic apparatus from carbohydrate-induced feedback inhibition (Selmar and Kleinwächter, 2013). Crucially, this secondary carbon diversion is not merely a passive overflow. Steroidal saponins act as potent non-enzymatic antioxidants, scavenging high-light-induced reactive oxygen species to stabilize thylakoid membranes (Erb and Kliebenstein, 2020). Consistent with this interpretation, leaf PS VII concentration covaried positively with *NPQ* in the exploratory PLS-SEM (**Figure 8B**), while *P_n_* was positively correlated with PS VII across treatments (**Figure 7B**). Together with the light-dependent accumulation of PS VII and the up-regulation of its biosynthetic genes (**Figures 6C, E, F**), these patterns suggest that enhanced saponin metabolism may contribute to both carbon allocation and photoprotection under high irradiance.

## 5 Conclusion

Increased growth irradiance revealed contrasting coordination between photosynthesis and carbon use in the two *Paris polyphylla* varieties. PPY maintained greater photosynthetic capacity at 400 μmol m^-^² s^-^¹ through increased Rubisco and RCA activities, *V_cmax_*, and *J_max_*, rather than through higher stomatal or mesophyll conductance. Its higher *ETR* and photorespiration rates, together with relatively low and stable *NPQ*, were consistent with greater use of absorbed energy in carboxylation and photorespiratory metabolism, while dark respiration remained low. PPC, in contrast, achieved its highest light-saturated photosynthetic rate when grown at 200 μmol m^-^² s^-^¹ and showed stronger *NPQ*, higher dark respiration, and a lower leaf SS/starch ratio under 400 μmol m^-^² s^-^¹. These photosynthetic contrasts were accompanied by differences in carbon pools and allocation. With increasing irradiance, PPY allocated more biomass belowground, increased the rhizome/leaf SS ratio, and accumulated more PS VII in leaves, together with stronger expression of two saponin-biosynthesis genes. Together, these results support coordinated regulation of the biochemical capacity for CO_2_ assimilation and downstream carbon utilization in PPY as growth irradiance increases. These responses are consistent with, but do not establish, a role for saponin biosynthesis as an additional carbon sink, a photoprotective mechanism, or both. Isotope tracing and direct measurements of saponin turnover and oxidative stress are needed to distinguish these possibilities under high irradiance.

## Data Availability

The original contributions presented in the study are included in the article/**Supplementary Material**. Further inquiries can be directed to the corresponding authors.
