# Supplementary material for: Contrasting photosynthetic capacity and carbon allocation under increasing irradiance in two Paris polyphylla varieties
Source: Front Plant Sci. 2026 Sep 4;17:1940365. doi: 10.3389/fpls.2026.1940365 (PMC13585735; doi:10.3389/fpls.2026.1940365)
Supplement: Supplementary file 1 [file SupplementaryFile1.docx]

**Table S1 Primers used in this study**

| **Primer name** | **Sequences (5' to 3')** |
| --- | --- |
| *ACT-RT-F* | GCTCCATCCTTGCTTCTCTTAG |
| *ACT-RT-R* | CTGAACACACCACCTCCTTTAT |
| *PpUGT80A2-F* | CAACAATGACGCAGTGAA |
| *PpUGT80A2-R* | CGTAGTCCATCGGAAGAG |
| *PpSmt2-1-F* | ACTGGAGGAACCATTTGTTGG |
| *PpSmt2-1-R* | GTCAGGTGCTGCGCTGTCT |

**Table S2** **Photosynthetic light response curve parameters in PPC and PPY under different light intensities**

| **Variety** | **Light intensity**  **(µmol m^-2^ s^-1^)** | ***AQY*** | ***P_nmax_***  **(µmol CO_2_ m^-2^ s^-1^)** | ***I_sat_***  **(µmol m^-2^ s^-1^)** | ***I_c_***  **(µmol m^-2^ s^-1^)** | ***R_d_***  **(µmol m^-2^ s^-1^)** |
| --- | --- | --- | --- | --- | --- | --- |
| PPC | 50 | 0.092 a | 3.83 b | 597.13 b | 4.89 b | 0.41 b |
|  | 200 | 0.089 a | 6.60 a | 676.58 a | 10.27 a | 0.82 a |
|  | 400 | 0.086 b | 5.66 a | 700.67 a | 11.58 a | 0.87 a |
| PPY | 50 | 0.079 a | 5.35 c | 643.36 c | 5.55 b | 0.46 a |
|  | 200 | 0.075 a | 7.43 b | 920.31 b | 5.39 b | 0.47 a |
|  | 400 | 0.067 b | 8.20 a | 1143.65 a | 6.07 a | 0.47 a |
| **ANOVA** |  |  |  |  |  |  |
| **Variety (V)** |  | <0.001 | <0.001 | <0.001 | <0.001 | <0.001 |
| **Light intensity (L)** |  | <0.001 | <0.001 | <0.001 | <0.001 | 0.023 |
| **V×L** |  | 0.002 | 0.001 | <0.001 | 0.001 | 0.037 |

Parameters were derived from light response curves for *P. polyphylla* var. *chinensis* (PPC) and *P. polyphylla* var. *yunnanensis* (PPY) grown under 50, 200, and 400 μmol m⁻² s⁻¹ light intensities. Variables include apparent quantum yield (*AQY*), maximum net photosynthetic rate (*P_nmax_*), light saturation point (*I_sat_*), light compensation point (*I_c_*), and dark respiration rate (*R_d_*). Data are presented as mean (n=3). Different lowercase letters indicate significant differences among treatments within each variety (*P* < 0.05). The bottom rows summarize two-way ANOVA *P*-values for the main effects of variety (V), light intensity (L), and their interaction (V×L).

**Table S3 Photosynthetic pigment concentrations in leaves of PPC and PPY under different light intensities**

| **Variety** | **Light intensity**  **(µmol m^-2^ s^-1^)** | ***Chl*** ***a***  **(mg g^-1^** **FW)** | ***Chl* *b***  **(mg g^-1^** **FW)** | ***Chl*** **(*a*+*b*)**  **(mg g^-1^ FW)** | ***Car***  **(mg g^-1^** **FW)** | ***Chl* *a*/*b*** |
| --- | --- | --- | --- | --- | --- | --- |
| PPC | 50 | 0.79 b | 0.44 a | 1.23 b | 0.28 b | 1.81 b |
|  | 200 | 0.90 a | 0.46 a | 1.36 a | 0.33 a | 1.98 a |
|  | 400 | 0.71 b | 0.36 b | 1.07 c | 0.27 b | 1.95 a |
| PPY | 50 | 0.58 a | 0.34 a | 0.93 a | 0.20 a | 1.69 b |
|  | 200 | 0.48 b | 0.27 a | 0.75 b | 0.18 b | 1.74 a |
|  | 400 | 0.32 c | 0.19 b | 0.52 c | 0.13 c | 1.71 ab |
| **ANOVA** |  |  |  |  |  |  |
| **Variety (V)** |  | <0.001 | <0.001 | <0.001 | <0.001 | <0.001 |
| **Light intensity (L)** |  | <0.001 | <0.001 | <0.001 | <0.001 | <0.001 |
| **V×**L |  | 0.001 | 0.003 | 0.002 | 0.003 | 0.002 |

Parameters include chlorophyll *a* (*Chl* *a*), chlorophyll *b* (*Chl b*), total chlorophyll (*Chl a*+*b*), carotenoids (*Car*), and the chlorophyll *a*/*b* ratio (*Chl* *a*/*b*) for *P. polyphylla* var. *chinensis* (PPC) and *P. polyphylla* var. *yunnanensis* (PPY) grown under 50, 200, and 400 μmol m⁻² s⁻¹ light treatments. Data are presented as mean (n=3). Different lowercase letters indicate significant differences among treatments within each variety (*P* < 0.05). The bottom rows summarize two-way ANOVA *P*-values for the main effects of variety(V), light intensity (L), and their interaction (V×L). FW, fresh weight.

**Table S4 Photosynthetic CO₂ response curve parameters in PPC and PPY under different light intensities**

| **Variety** | **Light intensity**  **(µmol m^-2^ s^-1^)** | ***CE***  **(mol m^-2^ s^-1^)** | ***P_nmax_***  **(μmol CO_2_ m^-2^ s^-1^)** | ***CSP***  **(μmol mol^-1^)** | ***CCP***  **(μmol mol^-1^)** | ***R_P_***  **(μmol m^-2^ s^-1^)** |
| --- | --- | --- | --- | --- | --- | --- |
| **PPC** | 50 | 0.018 a | 9.62 a | 2498.53 a | 109.17 a | 1.82 a |
|  | 200 | 0.024 a | 8.57 b | 1686.01 b | 105.05 b | 2.10 a |
|  | 400 | 0.022 a | 10.10 a | 1764.32 b | 107.77 a | 2.11 a |
| **PPY** | 50 | 0.008 c | 4.34 c | 2211.43 a | 103.64 b | 1.16 b |
|  | 200 | 0.016 b | 6.84 b | 1901.51 b | 88.68 b | 1.27 b |
|  | 400 | 0.040 a | 11.88 a | 1540.51 c | 163.74 a | 3.46 a |
| **ANOVA** |  |  |  |  |  |  |
| **Variety (V)** |  | <0.001 | <0.001 | <0.001 | <0.001 | <0.001 |
| **Light intensity (L)** |  | <0.001 | <0.001 | <0.001 | <0.001 | <0.001 |
| **V×L** |  | <0.001 | <0.001 | <0.001 | 0.001 | 0.001 |

Parameters were derived from intercellular CO₂ response curves for *P. polyphylla* var. *chinensis* (PPC) and *P. polyphylla* var. *yunnanensis* (PPY) grown under 50, 200, and 400 μmol m⁻² s⁻¹ light treatments. Variables include carboxylation efficiency (*CE*), maximum net photosynthetic rate (*P_nmax_*), CO₂ saturation point (*CSP*), CO₂ compensation point (*CCP*), and photorespiration rate (*R_P_*). Data are presented as mean (n=3). Different lowercase letters indicate significant differences among treatments within each variety (*P* < 0.05). The bottom rows summarize two-way ANOVA *P*-values for the main effects of variety (V), light intensity (L), and their interaction (V×L).
